# Editorial: Nutrition and mental health in the aging population

**DOI:** 10.3389/fpsyt.2023.1104762

**Published:** 2023-01-13

**Authors:** Yoram Barak

**Affiliations:** Dunedin School of Medicine, Department of Psychological Medicine, University of Otago, Dunedin, New Zealand

**Keywords:** nutrition-clinical, aging adults, mental health, depression, anorexia

Nearly 70 years ago, in 1954 a follow-up study of 577 San Mateo County residents over 50 years of age was carried out. Correlations between factors studied and morbidity were not conclusive, but suggested relationships between low economic status, low hemoglobin high caloric intake, low thiamine intake and low ascorbic acid intake and nervous system disease (1). Since then, over 50,000 studies focusing on nutrition and aging have been published. What have we learned?

Senotherapeutics are a class of drugs and natural products that delay, prevent, or reverse the senescence process—senolytics. Natural senotherapeutics from food sources—nutritional senotherapeutics—may constitute an interesting way to achieve better age-associated outcomes through personalized nutrition (2). This may read like wish-fulfilling science-fiction. But in order to advance our understanding of senolytics we need a vision. The articles in this Research Topic are a starting point.

*Nutrition, gut microbiota, and Alzheimer's disease* reviews the research that supports the role of intestinal microbiota in connection between nutritional factors and the risk for Alzheimer's disease onset and progression (Romanenko et al.). The *Perspective on chronic long-lasting anorexia nervosa* attempts to integrate the insufficient knowledge in this area and the impact of severe eating disorders on life course and brain health (Speciani et al.). A novel approach to the *Differential effects of sleep disturbance and malnutrition on late-life depression among community dwelling adults* demonstrates that while both sleep disturbance and malnutrition are significantly associated with late-life depression malnutrition may be more critically associated with depression in community-dwelling older adults (Hwang et al.). This may offer an insight into the mechanism through which late-life depression is a risk factor for dementia.

These articles demonstrate the complexity of interactions between nutrition and mental health. They will hopefully facilitate further research and clinical implications.

## Author contributions

The author confirms being the sole contributor of this work and has approved it for publication.
